# Clinical and paraclinical aspects of HIV infected patients with first-line antiretroviral regimens

**DOI:** 10.1186/1471-2334-13-S1-P7

**Published:** 2013-12-16

**Authors:** Ina Bîstrițchi, Tiberiu Holban, Constantin Spânu

**Affiliations:** 1 Department of Infectious Diseases, Tropical and Medical Parasitology, State Medical and Pharmaceutical University “Nicolae Testemițanu”, Chişinău, Republic of Moldova; 2 National Public Health Center, Republic of Moldova

## Background

We aimed to assess the clinical, immunologic and virologic evolution of naive HIV-infected patients, who were treated with first-line regimens of highly active antiretroviral therapy (HAART) during 1 year.

## Methods

The study included 149 adult patients diagnosed with HIV/AIDS infection (average age 36.28±0.81 years), supervised in the specialized department of the Clinical Hospital of Infectious Diseases “Toma Ciorbă”. The transmission of the HIV infection was in 87.25% (130) of the patients heterosexual and in 12.75% (19) by intravenous drug use. Out of the 149 patients who have initiated HAART, 94 (63.09%) were detected late with a number of CD4<350 cells/cmm, out of which 56 (37.58%) patients were detected very late with a number of CD4<200 cells/cmm. At HAART initiation, undetectable HIV RNA had 17 (11.41%) patients and HIV RNA>100,000 copies/mL had 46 (30.87%) patients. Out of all patients who received HAART during the first 48 weeks, 53 (35.57%) patients received AZT+3TC+EFV (regimen 1), 65 (43.62%) patients – AZT+3TC+NVP (regimen 2) and 31 (20.81%) patients – TDF+FTC+EFV (regimen 3). The late diagnosis is defined by the presence of AIDS associated diseases and/or the level of CD4<350 cells/cmm.

## Results

More than half (63.09%) of the investigated patients showed severe immunity suppression and/or clinical AIDS associated diseases. The most frequent AIDS related diseases were oropharyngeal candidiasis (55.03%), tuberculosis (22.82%), wasting syndrome (13.10%), herpes zoster (8.28%), Kaposi’s sarcoma (2.76%) and HIV encephalopathy (1.38%). Prior to initiation of HAART, 73.83% of the investigated patients were in AIDS stage (A3–10.07%, B3–27.52%, C2–6.71% and C3–29.53%). CD4 increase was for the first regimen from 189.54±13.25 to 290.39±21.13 cells/cmm, representing an increase from baseline 1.53-fold (p<0.001), for the second regimen – from 139.02±10.04 to 272.55±19.11 cells/cmm, an increase of 1.96-fold, which is a significant difference similar to the first regimen (p<0.001), and for the third regimen – from 163.42±22.4 to 256.87±68.33 cells/cmm, an increase of 1.57-fold (p>0.05).

## Conclusion

This study showed that more than half (63.09%) of HIV/AIDS – infected patients were detected late, with the number of T-lymphocytes CD4<350 cells/cmm, with or without AIDS related conditions. The therapeutic efficiency of HAART regimens in HIV infected patients was a success both immunologically and virologically, for all administered regimens, but an undetectable viral load and more sustainable increase of the CD4 count is higher (1.96 times) was determined for patients with second regimen (AZT+3TC+NVP).

